# Double suicide genes driven by kinase domain insert containing receptor promoter selectively kill human lung cancer cells

**DOI:** 10.1186/1479-0556-9-6

**Published:** 2011-03-22

**Authors:** Junrong Ma, Mi Li, Longyong Mei, Qinghua Zhou, Lunxu Liu, Xijie Yu, Guowei Che

**Affiliations:** 1 Laboratory of Endocrinology and Metabolism, West China Hospital, Sichuan University, P.R. China, 610041; 2 Department of Thoracic Surgery, West China Hospital, Sichuan University, P.R. China, 610041; 3 Tianjin Lung Cancer Institute; Tianjin Medical University General Hospital, Tianjin 300052, China

## Abstract

**Background:**

To investigate the selective killing efficacy of the double suicide genes driven by KDR promoter.

**Materials and methods:**

A double suicide gene system with the KDR promoter, pcDNA3-KDRp-CDglyTK, was constructed and transfected into lung cancer cell lines L9981 and NL9980, and human hepatocellular carcinoma cell line HepG2. The efficiency and specificity of the double suicide gene system were assayed by in vitro cellular proliferation and apoptosis, as well as in vivo xenograft studies.

**Results:**

The transgenic CD and TK genes were only expressed in L9981 and NL9980 but not in HepG2 cells. Pre-treating transfected cells with 5-Fc and GCV significantly reduced proliferation, enhanced apoptosis in L9981 and NL9980 but not in HepG2 cells. The tumor formed by L9981 and NL9980 cells with the double suicide gene system was much smaller in vivo.

**Conclusion:**

Tumor targeted expression of CDglyTK gene driven by KDR promotor represents a novel strategy for effective gene therapy of tumor with intrinsic KDR.

## Background

Tumor-specific targeting gene therapy is a widely used anti-tumor method. Regulated expression of a suicide gene with the promoters can primarily destroy tumor cells and leave the surrounding tissues undamaged. The a-fetoprotein promoter (AFP) is such a representative to activate an exogenous gene expression specifically in hepatocellular carcinoma (HCC) and has been applied to targeted gene therapy for HCC [1]. Nishino et al reported an approach to selectively kill c-Myc-expressing lung cancer cells by fusing the c-Myc gene promoter with TK gene [2].

Previous studies have shown that the KDR gene is specifically expressed in the vascular endothelial cells and some tumor cells. The expression level of KDR is correlated with the renewal rate of the vascular endothelial cells. The proliferation rate of the endothelial cells in tumor tissue is 500 times faster than that of the normal endothelial cells [3], which leads to the higher levels of KDR gene in many human tumor endothelial cells. We hypothesized that KDR promoter driven double suicide gene could be used as tumor-specific targeting approach to kill the tumor cells. A KDR promoter-driven double suicide gene (CDglyTK) expression system, pcDNA3-KDRp-CDglyTK, was constructed in the present study. Our research showed that this system could selectively reduce proliferation, enhance apoptosis, and reduce tumor formation in vivo in lung cancer cells.

## Materials and methods

### Cell lines

Human large cell lung cancer cells (L9981, NL9980), human umbilical vein endothelial cells (ECV304), and human hepatoma cells (HepG2) were obtained from Sichuan Provincial Key Laboratory of Lung Cancer Molecular Biology (Chengdu, China). Cells were cultured in RPMI-1640 medium with 10% fetal calf serum (FCS), 100 kU/L penicillin and 100 kU/L streptomycin at 37°C in a 5% CO_2 _incubator. Human lung adenocarcinoma cells (A549) were purchased from ATCC (USA) and maintained in RPMI-1640 medium with 10% FCS at 37°C in a 5% CO_2 _incubator.

### Intrinsic expression of KDR mRNA and protein in cancer cells

The mRNA expression of KDR was studied by reverse transcription-polymerase chain reaction (RT-PCR). Total RNA was extracted from cultured cells. RT-PCR was performed following vendor's instructions. β-actin mRNA was used as an internal control.

The protein expression of KDR was studied by Western blotting. The Cells were lysed in RIPA buffer. The blotting membrane was incubated overnight at 4°C with primary antibody: anti-KDR (1:1000 dilution; Cell Signaling Technology, Danvers, MA, USA), The blots were incubated for 1 h at room temperature with a horseradish peroxidase-conjugated secondary antibody (Chemicon, Temecula, CA, USA). Signals were visualized using ECL plus chemiluminescence substrate (Amersham, Piscataway, NJ, USA).

### Construction of pcDNA3-KDRp-CDglyTK plasmid vector

A 1.3 kb gene fragment encoding CD gene (Genebank S56903) and a 1.1 kb fragment encoding TK gene (Genebank V00470) were amplified from a CD and TK expressing vector pcDNA3-CDTK (a gift from the Laboratory of Medical Molecular Biology of Sichuan University, China) by standard polymerase chain reaction (PCR) techniques. The oligonucleotides CD-5' (5'-AAG CTT AGG CTA GCA ATG TCG AAT AAC GCT-3'), which introduced a Hind III site (underlined in the sequence) to the 5' end of the CD gene, and CD-3' (5'- GGA TCC TCC ACG TTT GTA ATC GAT GGC TTC-3'), which introduced a BamH I site (underlined in the sequence) to the 3' end and changed the TGA (stop codon) to GGA, were used as primers to amplify CD gene from pcDNA3-CDTK vector. The oligonucleotides TK-5' (5'- GGA TCC GGC GGG GGC GGT GGA GGA GGG GGT ATG GCT TCG TAC-3'), which introduced a BamH I site (underlined in the sequence) to the 5' end of the TK gene, and TK-3' (5'- TCT AGA TTA GTT AGC CTC CCC CAT CTC-3'), which introduced a Xba I site (underlined in the sequence) to the 3' end, were used as primers to amplify TK gene from pcDNA3-CDTK vector.

A 366 bp KDR promoter fragment was cloned from A549 cell genome by PCR, containing minimum core sequence of -125 to +227 of the KDR promoter (GeneBank KDR/flk-1 X89776). The primers used were: forward primer 5'-GCT CGA GTT GTT GCT CTG GGA TGT TCT-3' with a Nrul site at the 5' end, and reverse primer 5'-GAA GCT TGT GCC GGT AGG AGA GGA TAT-3' with a Hind III site at the 3' end.

The CD and TK gene fragments were cloned into the pcDNA3 vector, whose CMV promoter was replaced with KDR promoter. The nucleotide sequence for the pcDNA3-KDRp-CDglyTK vector was confirmed by DNA sequencing.

### Expression of pcDNA3-KDRp-CDglyTK system in cancer cells

The pcDNA3-KDRp-CDglyTK vector was transfected into L9981, NL9980, HepG2 cells by standard protocols. The transfected cells were cultured in the medium with G418 (400 ug/ml) to select out positive colonies. The expression of CD, TK, KDRp was confirmed by PCR and gel electrophoresis.

### In vitro study

#### Cell viability

The MTT approach was applied to determine the cellular viability. Cells were plated in 96-well plates at a density of 5000 cells/well overnight. After serum starvation for 24 h, cells were treated with 5 μg/ml GCV, 100 μg/ml 5-Fc, or 5 μg/ml GCV + 100 μg/ml 5-Fc, or 0.9% sodium chloride (physiological saline) as a control group. Then the cells were incubated with fresh medium containing 0.5 mg/mL MTT at the indicated time points. After 4-h incubation, medium was removed and purple blue sediment was dissolved in 150 μl DMSO. The relative optical density (OD) of each well was determined using a Bio-Rad 2550 EIA Reader (Bio-Rad, Hercules, CA, USA).

#### Apoptosis

Propidium iodide (PI) was used in flow cytometry analysis to assay apoptosis. Harvested cells were fixed with 4% paraformaldehyde and resuspended in 0.1% Triton-X-100 solution for 3 minutes. Cells were afterwards incubated in 0.01% RNase solution at 37°C for 30 minutes and then labeled with 0.05% PI for at least 30 minutes. Cells were assayed with EPISC-XL flow cytometry (Coulter USA) and analyzed with Multicycle software.

### Tumor cell xenograft

Tumor cells containing pcDNA3-KDRp-CDglyTK vector or empty vector were cultured under the same conditions. Dissociated cells were collected, rinsed thoroughly, and resuspended at 1 × 10^7 ^cells/ml in PBS. Four to six week-old male nude mice (purchased from Animal Center, Sichuan University) were anesthetized with isoflurane. A cell suspension (2 × 10^6 ^cells in 200 μl) was implanted subcutaneously. Mice were observed daily for the first 3 to 5 days post-operatively to assure the injection site was healthy. 125 mg/kg GCV and 1000 mg/kg 5-Fc were injected intraperitoneally daily from the 10^th ^day. Mice were closely monitored for the tumor burden. Mice were euthanized at 20 days after tumor injection. Tissue samples and other biological data were collected. All animal procedures were reviewed and approved by the Institutional Animal Care and Use Committee.

### Statistical Analysis

Statistical analysis was carried out using SPSS-10.0 software (SPSS, Chicago, IL). Measurement data were analyzed with Student's t test or f test and enumeration data with χ^2 ^test.

## Results

### Intrinsic expression of KDR in tumor cells

**Figure 1:**
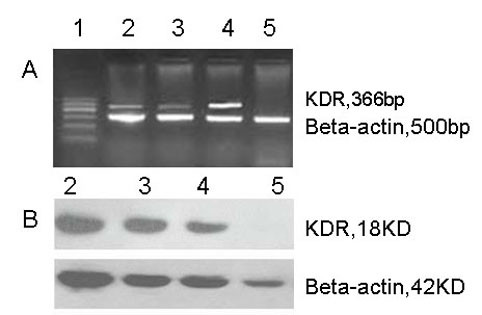
**Intrinsic expression of the KDR mRNA and protein**. mRNA (A) and protein (B) expression of the KDR was determined by RT-PCR and Western blotting, respectively. There was a significant amount of KDR expression in the NL9980, L9981 and ECV304 cells. The level of KDR expression in the HepG2 cells was not detectable. 1. DL2000 DNA marker; 2. NL9980 cells; 3. L9981 cells; 4. ECV304 cells (positive control); 5. HepG2 cells.

In order to select out suitable tumor cells as model to test the selective killing efficacy of the double suicide genes under regulation of the KDR promoter, KDR expression was studied by RT-PCR and Western blotting in human large cell lung cancer cell lines L9981 and NL9980 and a human hepatocellular carcinoma cell line HepG2. A human umbilical vein endothelial cell line ECV304, which is supposed to express intrinsic KDR, was used as a positive control. mRNA and protein expression of KDR was detected in NL9980 and L9981 cells (Figure 1). There was no KDR expression in HepG2 cells (Figure 1).

### External CD and TK gene expression in tumor cells

**Figure 2:**
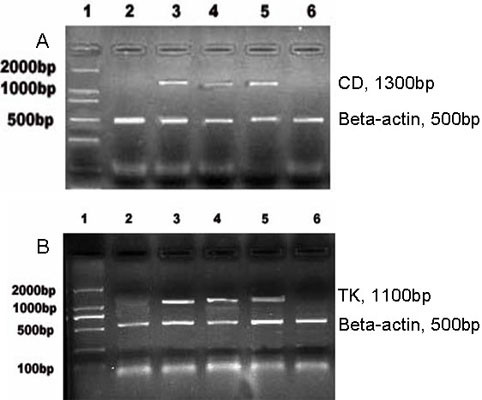
**Exogenous CD and TK mRNA expression**. The pcDNA3-KDRp-CDglyTK plasmid was transiently transfected into cells. The exogenous mRNA expression of CD (A) and TK (B) genes was determined by RT-PCR. The transgenic genes were only expressed in the L9981, NL9980 and ECV304 cells, but not in the HepG2 cells. 1. DL2000 marker; 2. Negative control; 3. L9981 cells; 4. NL9980 cells; 5. ECV304 cells (positive control); 6. HepG2 cells.

L9981, NL9980, HepG2 and ECV304 (positive control) cells were transfected with pcDNA3-KDRp-CDglyTK plasmid. To test whether the tansfected cells expressed suicide gene products CD and TK, RT-PCR was used to determine the CD and TK mRNA expression. As expected, CD and TK mRNAs were expressed only in L9981, NL9980, and ECV304 cells but not in HepG2 cells (Figure 2), indicating that the CD and TK genes were correctly regulated by the transgenic KDR promoter.

### In vitro cytotoxicity analysis of the KDRp/CD/TK gene-transfected cells

To determine the function of the exogenous CD and TK genes in tumor cells, the tumor cells with/without the pcDNA3-KDRp-CDglyTK system were treated with GCV and/or 5-FC, cellular survival rates were assayed with MTT method and calculated as the OD value of the pro-drug group/the OD value of the non-drug group × 100%. 5-Fc and/or GCV treatment did not change the cellular survival rate in HepG2 cells with or without CD and TK transgenes (Table 1). However, 5-Fc and/or GCV treatment significantly decreased the cellular survival rate in L9981 and NL9980 cells with CD and TK transgenes. The cells treated with 5-Fc and GCV together showed the maximal reduction in the cellular survival rate (Table 1).

**Table 1:** Cell survival rate in HepG2, L9981 and NL9980 cells with or without the double suicide gene system.

Cell line	Group	Cell survival rate (%)				*p*
		Control	GCV(5 ug/ml)	5-Fc(100 ug/ml)	GCV(5 ug/ml)+ 5-Fc(100 ug/ml)	
HepG2	HepG2	97 ± 5	96 ± 3	99 ± 1	95 ± 2	0.16
	HepG2-Vector	100 ± 3	97 ± 3	98 ± 1	99 ± 3	0.38
	HepG2-CDglyTK	99 ± 3	97 ± 6	94 ± 5	96 ± 3	0.57
	*p*	0.201	0.17	0.13	0.20	

L9981	L9981	96 ± 3	94 ± 2	95 ± 6	94 ± 4	0.08
	L9981-Vector	94 ± 3	95 ± 3	97 ± 5	95 ± 3	0.09
	L9981-CDglyTK	94 ± 5	67 ± 2	71 ± 4	34 ± 3	0.01
	*P*	0.32	0.002	0.0025	0.0021	

NL9980	NL9980	96 ± 1	96 ± 3	95 ± 2	97 ± 4	0.1
	NL9980-Vector	93 ± 3	94 ± 2	95 ± 4	95 ± 3	0.11
	NL9980-CDglyTK	96 ± 5	63 ± 1	66 ± 5	36 ± 4	0.00
	*P*	0.22	0.03	0.011	0.00	

The MTT method was used to determine cellular viability. 5-Fc and/or GCV treatment significantly decreased the cellular survival rate in L9981 cells with CD and TK transgenes. The cells treated with 5-Fc and GCV together showed the maximal reduction.

To further assay the function of the exogenous CD and TK genes in tumor cells, the tumor cells with/without the pcDNA3-KDRp-CDglyTK system were treated with GCV and/or 5-FC, cellular apoptosis was determined with flow cytometry analysis. Similar to the cellular survival results, 5-Fc and/or GCV treatment did not change the cellular apoptosis in HepG2 cells with or without the CD and TK transgenes (Table 2). However, 5-Fc and/or GCV treatment significantly enhanced the cellular apoptosis in L9981 and NL9980 cells with the CD and TK transgenes. The cells treated with 5-Fc and GCV together showed the maximal increase in apoptosis (Table 2).

**Table 2:** Cell apoptosis index in HepG2, L9981 and NL9980 cells with or wtihout the double suicide gene system.

Group	Apoptosis index(AI %)			*P*
		
	HepG2	HepG2-vector	HepG2-CDglyTK	
0.9% N S	6.3 ± 1.0	5.7 ± 0.3	6.3 ± 1.3	0.43
GCV(5 μg/ml)	7.1 ± 0.6	6.6 ± 0.7	7.2 ± 0.7	0.51
5-FC(100 μg/ml)	6.8 ± 0.7	7.0 ± 1.0	7.0 ± 0.9	0.16
GCV(5 μg/ml)+5-FC(100 μg/ml)	5.9 ± 0.5	6.1 ± 1.1	6.6 ± 1.1	0.18
*P*	0.07	0.12	0.34	

	L9981	L9981-vector	L9981-CDglyTK	
0.9% N S	5.1 ± 1.1	5.7 ± 1.3	5.4 ± 0.7	0.07
GCV(5 μg/ml)	5.5 ± 0.3	6.0 ± 0.3	9.7 ± 1.7	0.04
5-FC(100 μg/ml)	6.0 ± 0.8	6.7 ± 1.3	13.1 ± 2.7	0.031
GCV(5 μg/ml)+5-FC(100 μg/ml)	6.1 ± 0.2	6.1 ± 1.1	19.9 ± 4.2	0.026
*P*	0.19	0.17	0.012	

	NL9980	NL9980-vector	NL9980-CDglyTK	p
0.9% N S	6.0 ± 0.1	5.9 ± 0.3	5.8 ± 1.7	0.12
GCV(5 μg/ml)	6.5 ± 0.2	6.3 ± 1.3	11.2 ± 2.4	0.03
5-FC(100 μg/ml)	6.1 ± 0.8	5.7 ± 0.8	12.9 ± 3.6	0.024
GCV(5 μg/ml)+5-FC(100 μg/ml)	5.9 ± 0.3	6.1 ± 1.5	23.1 ± 5.0	0.021
*P*	0.11	0.11	0.0092	

Cells were stained with popidium iodide and analyzed with flow cytometry to detect apoptosis. 5-Fc and/or GCV treatment did not change the cellular apoptosis in the HepG2 cells with or without CD and TK transgenes.

### In vivo tumor formation

**Figure 3:**
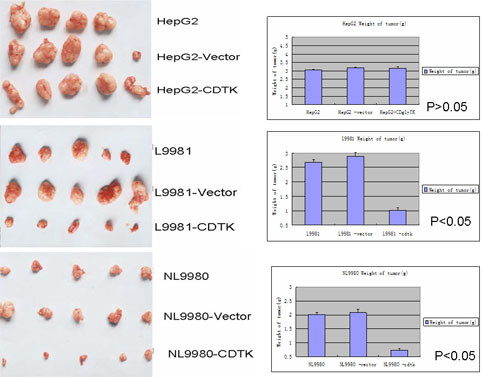
**In vivo tumor formation by HepG2 (A), L9981 (B) and NL9980 (C) cells**. HepG2, L9981 and NL9980 cells with different pre-treatments were implanted subcutaneously into nude mice. Ten days after implantation, the mice were treated with 5-Fc and GCV for another 20 days. The mice were sacrificed and the tumors were removed intact and weighed. There were no significant difference in tumor weights among different treatment of HepG2 group, but L9981 and NL9980 groups with CD and TK transgene showed significantly smaller tumors than the other two groups.

To further assay the efficacy of the double suicide genes under regulation of the KDR promoter, L9981, NL9980 and HepG2 cells with pcDNA3-KDRp-CDglyTK system or empty vector were implanted subcutaneously into nude mice. Ten days after implantation, the mice were treated with 5-Fc and GCV intraperitoneally for another 20 days. The mice were sacrificed and the tumors were removed intact and weighed. There were no significant difference in tumor weight and size in HepG2 cells, no matter of different treatment and with or without the pcDNA3-KDRp-CDglyTK system (Figure 3A). In the mice implanted with L9981 and NL9980 cells, the tumor cells with the CD and TK transgenes formed significantly smaller tumors than the tumor cells without the CD and TK transgenes (Figure 3B and 3C).

## Discussion

In the present study, the KDR promoter-driven CD/TK double suicide gene system was successfully constructed. CD/TK gene expression was only detected in human large cell lung cancer cell lines L9981 and NL9980, which expressed intrinsic KDR, but not in the human hepatocellular carcinoma cell line HepG2, which did not express intrinsic KDR. The present data also indicate that KDR promoter is regulated by its natural elements. The core regulatory region between -225 bp to 125 bp of the KDR promoter has been successfully cloned and testified by DNA sequencing [4]. Because of its specific expression in the tumor tissues, KDR promoter has been used to express target genes in some tumors. Modlich et al [5] used the KDR promoter to regulate TNF-α expression in tumor vascular endothelial cells (TVEC). Szary et al [6] successfully introduced a KDR promoter-regulated CD gene into murine sarcoma cells and human ovarian cancer cell line OVP10. A KDR promoter-driven CD/TK plasmid pcDNA3-KDRp-CDglyTK was constructed and introduced into the lung cancer cell lines with different KDR expressing levels. The stable expression of CDglyTK in the cell lines with higher intrinsic KDR levels indicates that the cloned KDR promoter was regulated by its intrinsic regulatory elements.

In vitro experiments showed that the double suicide genes were functionally only in the L9981 and NL9980 cells. The treatment with 5-FC, GCV, or 5-FC+GCV showed no notable difference in the cell survival rate among HepG2, HepG2-vector, and HepG2-CDglyTK cells, which indicates the transgenic CDglyTK genes did not express double suicide gene because of the inactivity of KDR promoter in the HepG2 cells. On the other hand, single pro-drug treatment with either 5-FC or GCV significantly decreased cell survival rate in the CDglyTK transgenic groups in the KDR-expressing cell lines (L9981 and NL9980). Previous results indicated higher killing efficiency of the combined suicide gene system than any single system, due to the synergetic cytotoxicity of the combined gene system. Rogulski et al [7] reported that the CDglyTK-transducted neuroglioma cells were easier to be extinguished. The radiation sensitivity of the CDglyTK-expressing cells was notably high, reaching 2.44 times of the CD/5-FC single system and 3.90 times of the BVdU/5-FC single system. The increased pro-drug sensitivity, due to the effect of double suicide genes, can overcome the insensitivity of the HSV-TK/pro-drug resistant cells in the recurrent tumors. At the same time, it can reduce the dosage of pro-drug treatment and increase the radiation sensitivity of the target cells [8]. The combined treatment of 5-FC and GCV resulted in a lower cell survival rate than single pro-drug treatment, which indicates the enhanced killing effect of the combined pro-drug treatment.

Our in vivo xenograft experiments showed tumors from the lung cancer cells were significantly suppressed by the systemic treatment of pro-drug 5-FC and GCV. 5-FC and GCV showed higher suppressing effect on the tumors from the highly metastatic human large cell lung cancer cell line L9981 than that of less metastatic human lung cell line NL9980, while no effect was shown on the tumors from the hepatic carcinoma HepG2 cells. These results further confirmed the efficacy of the double suicide genes under regulation by the KDR promoter.

Our results indicate the double suicide genes regulated by the KDR promoter can be specifically expressed in the KDR-expressing cells such as human lung cancer cells. 5-FC and GCV treatment shows satisfactory drug synergism as the killing effect of the combined 5-FC+GCV treatment is significantly higher than that of any single pro-drug treatment.

## Conclusions

Our work suggests that the KDR promoter is capable of regulating a double suicide gene system in human lung cancer cells, thus providing laboratory evidence to develop a gene therapy approach against various cancers. Our research indicates that expression of CDglyTK genes under the control of KDR promotor represents a new strategy for effective gene therapy of tumors expressing intrinsic KDR.

## List of abbreviations

KDRp: Kinase domain receptor promoter; GCV: Ganciclovir; 5-Fc: 5-Fluorocytosine; 5-Fu: 5-Fluorouracil; HSV-tk: Herpes simplexvirus-thymidinekinase; CD: E.coli-cy-tosinedeaminase;

## Competing interests

The authors declare that they have no competing interests.

## Authors' contributions

JRM and GWC carried out all the experiments, analyzed results and drafted the manuscript. ML and LXL helped to edit the manuscript. Some help was given by LYM in analysis of data and preparation of the manuscript. XJY participated in the design of the study and the critical view of manuscript writing. All authors read and approved the final manuscript

## Acknowledgements

The authors would like to thank Dr. JinFeng DING for kindly providing the CD and TK plasmid; this work was supported by the National Natural Science Foundation of China (No.30872547, to Guowei Che).
